# Correcting for Visuo-Haptic Biases in 3D Haptic Guidance

**DOI:** 10.1371/journal.pone.0158709

**Published:** 2016-07-20

**Authors:** Femke E. van Beek, Irene A. Kuling, Eli Brenner, Wouter M. Bergmann Tiest, Astrid M. L. Kappers

**Affiliations:** MOVE Research Institute Amsterdam, Department of Human Movement Sciences, Vrije Universiteit Amsterdam, Amsterdam, The Netherlands; Emory University, UNITED STATES

## Abstract

Visuo-haptic biases are observed when bringing your unseen hand to a visual target. The biases are different between, but consistent within participants. We investigated the usefulness of adjusting haptic guidance to these user-specific biases in aligning haptic and visual perception. By adjusting haptic guidance according to the biases, we aimed to reduce the conflict between the modalities. We first measured the biases using an adaptive procedure. Next, we measured performance in a pointing task using three conditions: 1) visual images that were adjusted to user-specific biases, without haptic guidance, 2) veridical visual images combined with haptic guidance, and 3) shifted visual images combined with haptic guidance. Adding haptic guidance increased precision. Combining haptic guidance with user-specific visual information yielded the highest accuracy and the lowest level of conflict with the guidance at the end point. These results show the potential of correcting for user-specific perceptual biases when designing haptic guidance.

## Introduction

In remote, dangerous or space-limited environments, teleoperation systems show great potential. In these systems, the operator uses a master device to control a slave system, which is placed in the environment. The master and the slave can be separated over a large distance. A consequence of the separation between operator and environment is the lack of natural feedback to the operator, so these systems are usually equipped with devices to provide haptic feedback in such a way that it resembles natural haptic feedback [1]. In addition to recreating the natural scene, these feedback devices could also be used to augment reality, of which the concept of haptic guidance is an example. In haptic guidance, forces inform the operator about the task objective by guiding the operator towards the target location [2]. This concept can, for instance, be used in a peg-in-hole task, in which a peg needs to be placed at a certain position and orientation to allow it to enter the hole. To guide the user to the correct position, haptic guidance in the form of an attractive force field, which has zero force at the desired position, can be used. One of the big challenges is to design these guidance forces in such a way that they are easy in use. In order to do this, knowledge on human perception is essential. One of the potential problems could be that human perception is not veridical, as is evident from various biases within and between modalities. Perceptual biases can be consistent across participants, such as the radial-tangential illusion [3], or they can be consistent within, but not between participants, such as the user-dependent error-patterns in the perception of force direction [4]. In a natural situation, such as trying to grasp an object, humans automatically correct for such biases, for instance by using both visual and haptic information. In teleoperation situations, this automatic correction might not be possible, for instance because operators do not see their hands while performing actions and thus cannot align their visual and haptic information. For haptic devices, it has been shown that correcting the mapping between operator and slave movements, by using parameters that are consistent across participants, increases user performance [5]. The question in our study was if it is also useful to correct for user-specific biases in the design of haptic guidance, by making user-specific adjustments.

To test this question, we used the well-known paradigm of visuo-haptic biases [6]. When participants are asked to bring their unseen hand to a visual target position, they make large errors, which are called visuo-haptic biases. These errors are idiosyncratic: they are consistent within, but not between participants [7–9]. Even when these biases are measured a month later, they are still consistent [10] and they are not affected by imposed forces or torques [11, 12]. When the targets are restricted to a horizontal plane, the biases are larger when the arm is further away from the body along the body midline [13, 14] or when the targets are positioned further to the left or right [15]. These natural mismatches are far less likely to be corrected for by the nervous system than are externally imposed mismatches [16].

We used the existence of visuo-haptic biases to test for the effectiveness of user-specific adjustments of haptic guidance, in a task in which participants were asked to localize visually presented targets without seeing their hand. When perception is biased, there is a difference between the physical and the perceived properties of a stimulus. In the case of visuo-haptic biases, there is a difference between the visual perception of the location of the stimulus and the haptic perception of that location. The practical implication of this is that if haptic guidance is directed towards a visual target, participants do not perceive it to be directed to that location. If the biases are known, it is possible to correct for them by adjusting the feedback information. In this way, the visual and haptic information could be made perceptually consistent, rather than physically consistent.

**Fig 1:**
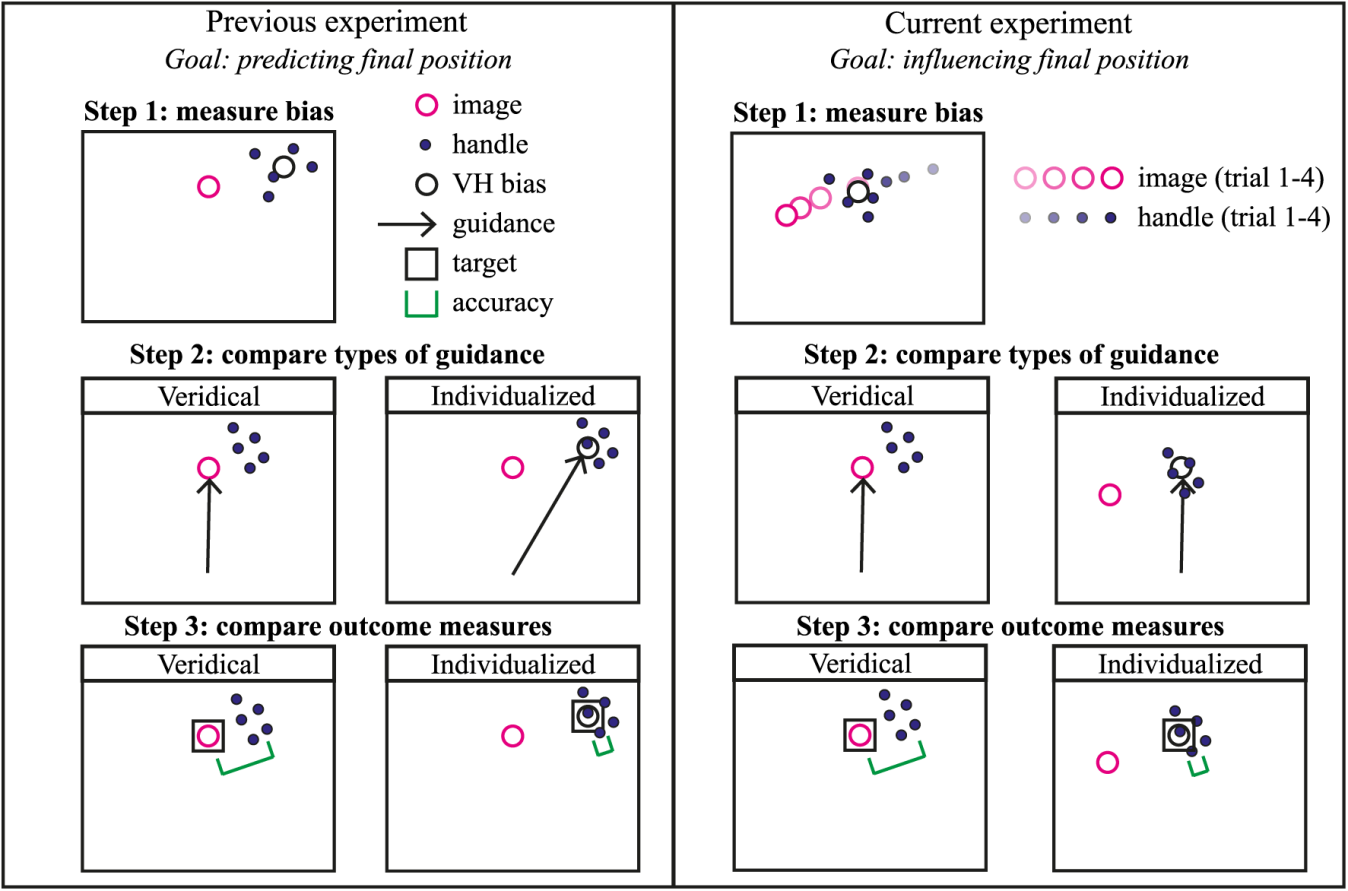
Illustration of the protocol of the previous and current experiment. In the previous experiment (left box), the goal was predicting where participants would position the handle, when being asked to move to a position. In order to do that, the direction of the guidance force was adjusted, while the position of the visual image was constant throughout the experiment. The outcome measure was how closely the mean end position of the handle matched the center of the guidance force field. In the current experiment (right box), the goal was influencing where participants would position the handle. To achieve that, the visual image position was shifted to match the visuo-haptic bias, while the guidance force was always directed towards the original visual position. The outcome measure was how closely the mean handle position matched the original visual image position (and thus the center of the guidance force field).

In a previous experiment, it was found that correcting for visuo-haptic biases in a 2D situation improved operator performance [17]. In the current study, we used the same task of pointing to visual targets with the unseen hand. There are two important differences between the previous study and the current one, which are illustrated in Fig 1. These differences are: 1) in the previous study, the concept was investigated in 2D, whereas we used a 3D setup in the current study; and 2) in the previous study, the information was corrected by changing the position of the haptic guidance, while in the current study, we adjusted the position of the visual information. So, in the previous experiment, the visual image was always presented at the same position, and veridical haptic guidance, towards the target position, was compared to haptic guidance towards the target position shifted by the measured bias. In the current study, we shifted the visual image, while the haptic guidance and the haptic target position were constant. In both methods (shifting the guidance position or shifting the position of the visual information), the aim was to reduce the amount of conflict between the modalities. The approach was somewhat different: in the previous experiment, participants reached the target position shifted by the visuo-haptic bias and not the original target position when performing consistently. When shifting the visual image, which was done in the current experiment, participants do reach the target position when performing consistently.

In summary, the aim of this study was to test the effectiveness of user-specific adjustments in haptic guidance, for which we used the paradigm of visuo-haptic biases in a 3D situation. We first measured the visuo-haptic bias and then compared three conditions: 1) a condition with visual images that were adjusted to the user-specific bias, without haptic guidance, 2) a condition with veridical images, combined with haptic guidance, and 3) a condition with shifted visual images, combined with haptic guidance.

## Materials and Methods

### Participants

Twelve participants took part in the experiment. The calibration failed for one participant, which we only discovered upon completion of the experiment. Therefore, the data of that participant could not be used. The data of the other eleven participants were used in the analysis. Three participants were males and eight were females, nine were right-handed and two were left-handed. Their age was 30±3 years (mean±standard deviation). None of the participants had a history of neurological disorders, all had normal or corrected-to-normal visual acuity, and none were stereo blind. Prior to the experiment, they received written instructions and signed an informed-consent form. The experiment was approved by the Ethics Committee of the former Faculty of Human Movement Sciences (ECB).

### Apparatus

**Fig 2:**
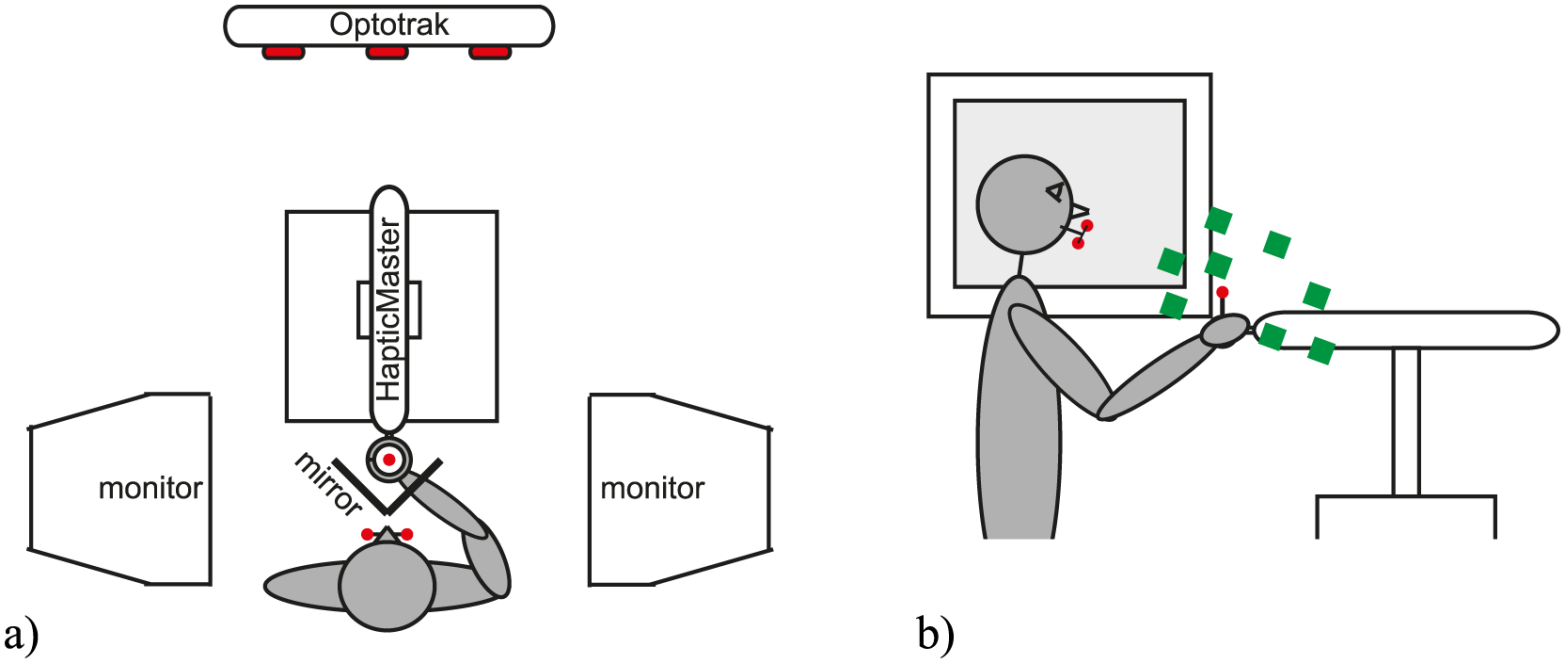
Participant in the setup. The participant was seated between 2 monitors, which projected images onto the semi-silvered mirrors to present target cubes in 3D. The participant used the handle of the HapticMaster to point at the perceived target locations. In some conditions, the HapticMaster exerted guidance forces, while in other conditions, it only passively followed the movements of the participant. The position of the handle and the head orientation of the participant were tracked using the Optotrak system (cameras are shown in red and markers are shown as red dots). a) Top view. b) Side view with target locations depicted as green cubes. For the precise coordinates of the target positions, see Table 1.

The setup was a combination of a visual display to provide visual information in 3D, a haptic device to provide guidance forces and a position tracking system, as shown in Fig 2. The visual display consisted of two CRT monitors (1096 × 686 pixels, 47.3 × 30.0 cm, refresh rate 160 Hz), which projected images onto two mirrors in front of the participant. By providing a different image for each eye, a 3D image could be created. Sousa et al. [18] provide a more elaborate description of the visual setup. Participants were seated on a height-adjustable chair to make sure that the mirrors were positioned at eye-height. The room was completely dark except for the light from the screens.

An Optotrak system (type 3020, Northern Digital Inc.) was used to track the position of 4 Infra-Red Emitting Diodes (IREDs) with a frequency of 250 Hz. Three IREDs were attached to a bite-board, and one IRED was attached to the handle of the haptic device. During the experiment, participants wore the bite-board in their mouth. Prior to the experiment, we determined the position of the eyes relative to this bite-board, using a procedure described previously in Sousa et al. [19]. During the experiment, the markers on the bite-board were tracked constantly by the Optotrak in order to display correct images to the participants, even when they made head movements. In this way, the 3D information was realistic, while allowing participants to move their head during the experiment. The fourth IRED was used to track the position of the handle of the haptic device in the Optotrak coordinate frame. Since the orientation of the handle could not change, we could use a single marker to determine the position of the center of the handle.

The haptic device was a HapticMaster (Moog Inc.), a strong admittance-controlled haptic device, which can also record position and force data [20]. In this experiment, the device recorded force and position data with a sampling frequency of 256 Hz. Its inertial mass was set to 3 kg throughout the experiment, and it moved frictionlessly through its closed-loop control. In the conditions without haptic guidance, the device followed the movements of the participants passively, while only compensating for its own weight. In the other conditions, it exerted a guidance force on the hand of the participant. Participants held the ball-shaped handle by enclosing it with their dominant hands. Their non-dominant hand was used to press two buttons to proceed to the next trial. Prior to the experiment, the coordinate systems of the Optotrak and the HapticMaster were aligned.

### Protocol

The experiment consisted of four conditions. All participants started with the same condition, while the other three conditions were presented in counterbalanced order. In all conditions, visual targets were presented using small green cubes (1 × 1 × 1 cm). The task for the participant was always the same: align the center of the ball-shaped handle of the HapticMaster with the center of the visual target cube. The position of the handle of the HapticMaster was never visible. When the participants were satisfied with the alignment of the centers, they pressed two spacebars with two fingers of their non-dominant hand to confirm the position in both measurement systems. After pressing the spacebars, a new trial started automatically from the end position of the previous trial. Participants were told that in some conditions, the haptic device would exert forces to help them reach the target. They were instructed that these forces would always help them to move in approximately the right direction, and that they would not always be correct when being close to the target. It was stressed that their task was to indicate the visible target’s position as accurately as possible, irrespective of the direction of the forces. In all conditions, 8 target positions were used, which are shown in Fig 2b and in Table 1. Each target position was repeated 20 times per condition, resulting in 160 trials per condition. A pseudo-random order was used for target presentation: the trials were divided into blocks, in which each target position was presented once. Within these blocks, the order was randomized. The same target was never presented on two consecutive trials. The 20 blocks of each condition were presented consecutively, without breaks.

**Table 1:** Target positions, with respect to the middle of the workspace.

Target	x [cm]	y [cm]	z [cm]
1	-7.5	-1.5	-6.0
2	2.5	1.5	6.0
3	7.5	4.5	-2.0
4	-2.5	4.5	2.0
5	2.5	-4.5	-2.0
6	-7.5	1.5	2.0
7	7.5	-4.5	-6.0
8	-2.5	-1.5	6.0

In the first condition (VA: constantly adjusting visual information only), the aim was to find the visual image position (${\vec{x}}_{v,k}$) of target *k* (*k* = 1, 2, …, 8) at which the position of the haptic representation (${\vec{x}}_{h,k}$) of that image coincided with the intended target position (${\vec{x}}_{t,k}$). To achieve this, the position of the visual image was adjusted on trial *n* (*n* = 1, 2, …, 20) based on the measured visuo-haptic bias. On the first trial, the visual target was presented at the target position (${\vec{x}}_{t,k}$). Participants placed the handle at their haptic representation of the visual image position (${\vec{x}}_{h,k,n}$), which was usually different from the actual target position. The error between the haptically perceived position and the target position was calculated and the visual image position was adjusted in the next trial for each target position (*k*) separately, using:

$$\begin{array}{c} {\vec{x}}_{v,k,n+1}={\vec{x}}_{v,k,n}-0.4\left({\vec{x}}_{h,k,n}-{\vec{x}}_{t,k}\right). \end{array}$$ (1)

**Fig 3:**
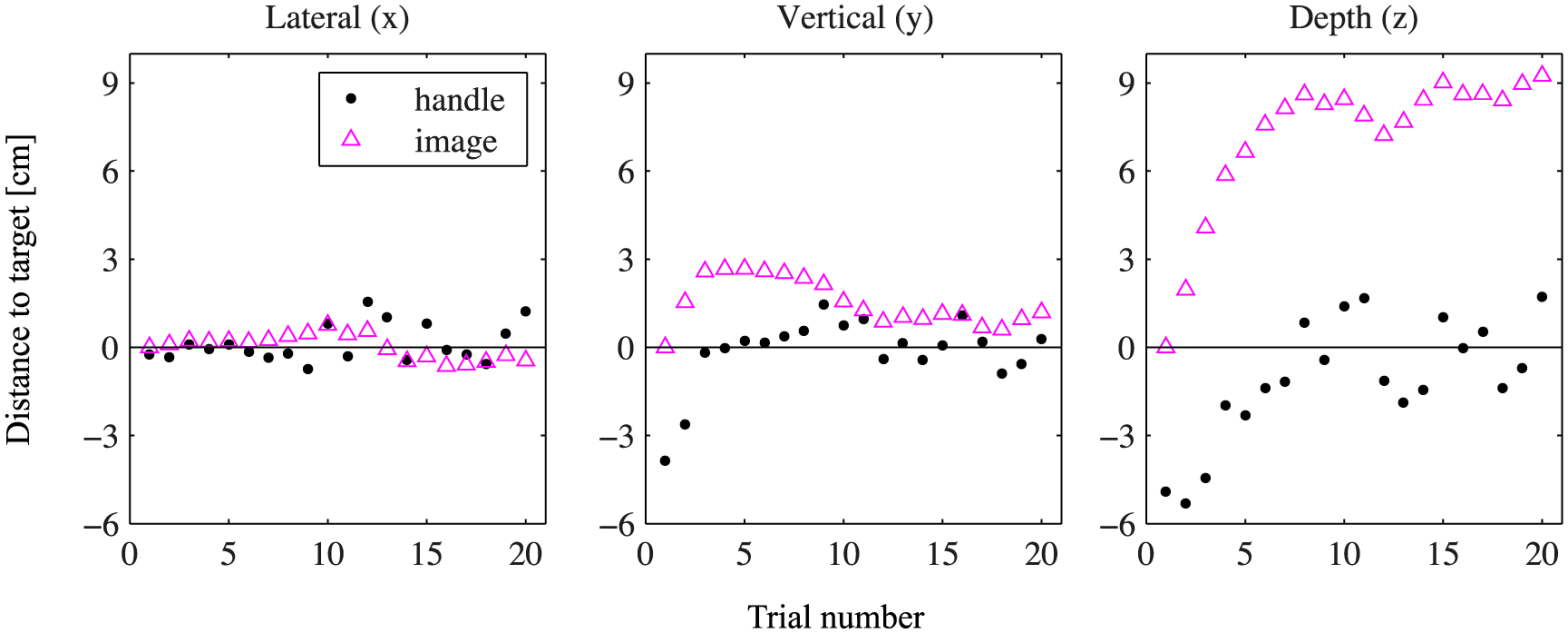
Illustration of the adaptive procedure used in condition VA. The magenta triangles show the positions of the visual image, which is initially presented at the target position, and the black dots indicate the end positions of the device handle. In all directions, zero indicates the target position. The three panels show the projections of the data onto the cardinal axes for one target. The visual image is shifted so that the end positions of the handle will be closer to the target at the last trials. The mean position of the visual images in trials 11 through 20, which is fairly stable with some noise around the mean, was used as the position of the shifted visual image throughout the rest of the experiment.

In this way, the visual image (${\vec{x}}_{v,k,n}$) was adjusted on each next trial (${\vec{x}}_{v,k,n+1}$) in the direction opposite to the error between the end point of the participant’s movement in the current trial (${\vec{x}}_{h,k,n}$) and the target position (${\vec{x}}_{t,k}$). As a result, the participant should be placing the handle at the target position (${\vec{x}}_{t,k}$), after some trials. This allowed us to determine the visual position that corresponded perceptually with the intended haptic target position. The adaptive value of 0.4 was chosen, as this is an optimal parameter for some such adaptive tasks [21]. A typical example of data from this condition is presented in Fig 3.

**Fig 4:**
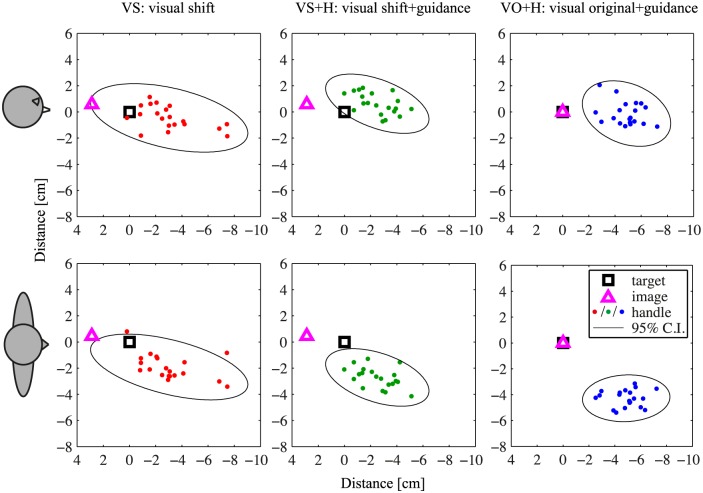
Typical examples for conditions VS, VS+H, and VO+H for one target and one participant. The black square shows the target position. The magenta triangle shows the position of the visual image. The dots indicate the end positions of the handle. The ellipse is a 2D representation of the 95% confidence ellipsoid. The first row shows a side view (i.e. horizontal axis shows depth direction, vertical axis shows vertical direction), and the second row shows a top view of the data (i.e. horizontal axis shows depth direction, vertical axis shows lateral direction). First column: typical example of condition VS. The visual image is shifted from the target position. The target square lies inside the confidence ellipsoid, but the ellipsoid is fairly large. Second column: typical example of condition VS+H. The visual image is again shifted, and haptic guidance towards the target position is added. The target square is still fairly close to the confidence ellipsoid. The volume of the ellipsoid is smaller than in VS. Third column: typical example of condition VO+H, in which the visual image is shown at the target location, while haptic guidance towards the target position is also added. Again, the volume of the confidence ellipsoid is fairly small, but now the ellipsoid is much farther from the target than in VS+H.

In the second condition (VS: shifted visual information only), the means of the visual image positions of the last 10 trials for each target position of condition VA were used as the visual image positions. The aim of this condition was to measure if these shifts of the visual image positions indeed resulted in participants indicating their end positions close to the target positions. Moreover, the perceptual precision could be determined in this way. A typical example of data from this condition is presented in Fig 4 (left column).

In the third condition (VS+H: shifted visual information and haptic guidance towards the target location), haptic guidance forces were added to the situation in condition VS. These guidance forces were designed as an attractive force field, with a linear stiffness of 50 N/m, and with an upper limit of 3.5 N to avoid large forces. So, at the target position, the guidance force was 0, while the force towards the target increased linearly with the distance from the target. In this condition, the visual image and the position of the minimum of the guidance force field were not presented at the same physical position, but the shifted visual position was used to correct for the visuo-haptic bias. A typical example of data from this condition is presented in Fig 4 (middle column).

In the fourth condition (VO+H: veridical visual information and haptic guidance towards the target location), haptic guidance forces were the same as in condition VS+H. The visual image was also presented at the target location, so all information was presented veridically. A typical example of this condition is presented in Fig 4 (right column).

In between the conditions, participants took a break as long as they preferred, in which they were allowed to take the bite-board out of their mouth. Each condition took about 8 minutes, so the total experiment lasted about 45 minutes per participant.

### Data analysis

To analyze the data, 95% confidence ellipsoids were fitted to the end points of the participant’s movements, for each target, condition and participant. To assess the accuracy of the participant’s performance, the distances between the centers of the ellipsoids and the actual target positions were calculated. Hence, a larger number indicates a lower accuracy. These values were averaged across target positions prior to statistical analyses. A similar calculation was done for condition VA, but here the difference between the target position and the mean position of the images in the last 10 trials of condition VA was used, since this represents the visuo-haptic bias. So, this value indicates how far the visual image needed to be shifted in order for the participant to perceive the hand at the target location. The target locations, shown in Table 1, were only used as the visual image positions in condition VO+H and in the first trial of condition VA. In all other conditions, participants were asked to move towards a shifted visual image, while the accuracy measure was always based on how closely the mean end positions matched with the target locations.

To assess the precision of the participant’s performance, the volumes of the ellipsoids were used, so a larger number indicates lower precision. Again, these values were averaged across target positions prior to performing statistical analyses. A repeated measures ANOVA was performed for both accuracy and precision data separately, on conditions VS, VS+H, and VO+H, to assess the effect of condition. Post-hoc tests with Bonferroni correction were used to compare all possible pairs of conditions. For all repeated measures ANOVAs, Greenhouse-Geisser correction was used when the sphericity-criterion was not met according to Mauchly’s test.

The level of conflict between the haptic guidance and the participant’s judgement was assessed by calculating the amount of guidance force that was still present when participants decided they had reached the target position. This measure is comparable to the accuracy measure, but the guidance force was limited to a maximum of 3.5 N, so, the distance measure was not limited, while the conflict measure had a limit of 3.5 N. It also provides an additional way of looking at the data, as this measure directly shows how much force was left on the handle when the participant decided (s)he had reached the end position. This measure was only calculated for the conditions in which there was haptic guidance, VS+H and VO+H, and the difference in this measure between the conditions was tested with a paired *t*-test.

The directions of the errors in the different conditions were assessed by comparing them to the directions of the visuo-haptic biases measured in condition VA. This was done by calculating, for each condition, the length of the projection of the error on the vector measured in condition VA. The ratio between the projected vector and the error vector was calculated to assess the amount of alignment between the two. Thus, a ratio of 1 means that the two vectors were oriented in exactly the same direction, a value of 0 means that they were oriented perpendicularly, and value of -1 means that the vectors were oriented in exactly opposite directions. The reason for calculating these values was to assess if the errors measured in the different conditions were related to the visuo-haptic biases. We expected that to be the case for VO+H, but finding a similar relation for VS+H would mean that the adaptation procedure in VA had not been completely successful.

Prior to the analyses, an outlier analysis was performed. For each target, participant, and condition, end points that were more than 4 standard deviations away from the mean of the data, when the mean was calculated without that particular end point, were considered to be outliers. This resulted in a total of 29 outliers, which was 0.4% of the data. Some of the outliers were caused by a mismatch between the two measurement systems, when participants accidentally had not pressed both space bars to proceed to the next trial. These mismatches were usually noticed quickly by the participants, since the guidance force was directed towards the wrong target if a mismatch occurred. For one participant, the visual image presented in the first condition was shifted in such a way that it was not visible any more for two of the targets, because it had crossed the limits of the monitors. For that participant, the data of those two targets were not used in the analysis.

## Results

**Fig 5:**
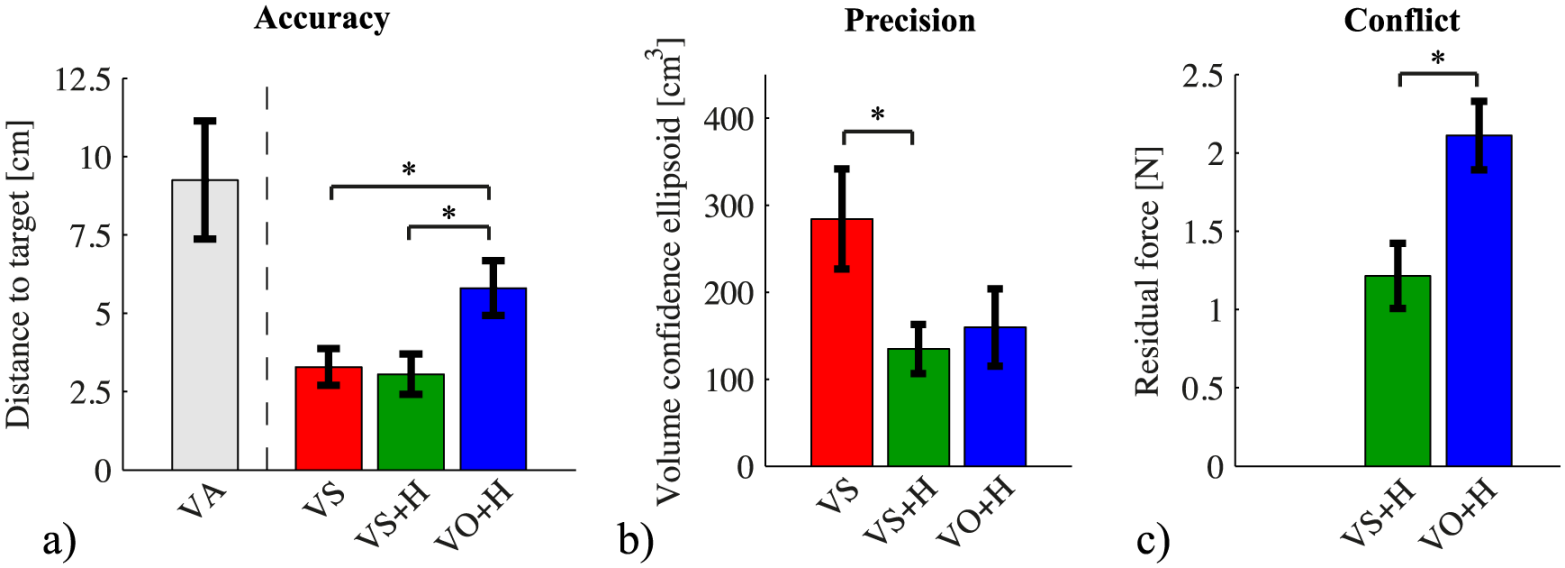
Accuracy, precision and amount of conflict. All errors bars show mean±one standard error. * Significant difference in post-hoc comparisons. a) Accuracy of responses: the distance between indicated and physical target position. Data of the last 10 trials for each target in condition VA (adjusting visual information) are presented as a reference, showing the difference between the physical target position and the position of the visual image. Note that presentation of a shifted visual image (VS and VS+H) yields the highest accuracy. b) Precision of responses: the volume of the 95% confidence ellipsoid. Adding haptic guidance increases the precision of the responses (VS+H and VO+H). c) Conflict: the residual force that was still present on the handle when the participants indicated they had reached the target. The level of conflict is lower for the shifted visual image.

**Fig 6:**
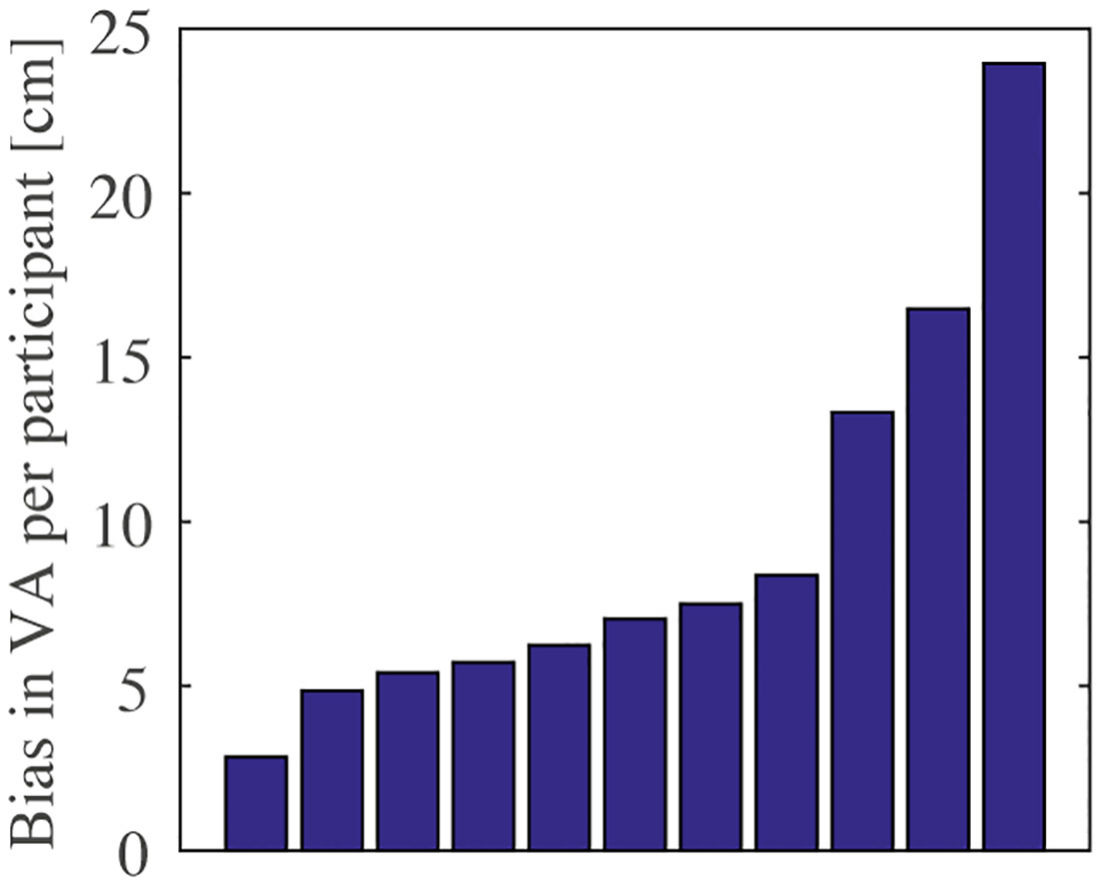
Magnitude of the visuo-haptic biases per participant, as measured in condition VA. Each bar represents one participant, and bars are ordered according to bias magnitude. Note that there is a wide range of biases across participants.

The accuracy of the responses is shown in Fig 5a. For condition VA, in which the visual positions were adjusted according to the participant’s responses, the mean difference between the target position and the positions of the visual image in the last 10 trials of each target position indicates the size of the visuo-haptic bias, which was 9.3±1.9 cm (mean±standard error). The visuo-haptic biases were mostly in the depth direction, as can also be seen in the typical example in Fig 4. The size of the bias and the presence of components other than the one in the depth direction were different between participants, as can bee seen in Fig 6. For the conditions other than VA, the distance between the mean target locations indicated by the participants and the target locations were 3.3±0.6 cm for VS, 3.1±0.6 cm for VS+H, and 5.8±0.9 cm for VO+H. The repeated measures ANOVA showed a significant main effect of condition (*F*_1.2,12_ = 19, *p* < 0.001). Bonferroni-corrected post-hoc comparisons showed that VO+H differed significantly from both VS (*p* < 0.0035) and VS+H (*p* < 0.0031), while VS and VS+H did not differ significantly (*p* = 1.0). In conditions VS and VS+H, in which the shifted visual image was presented, the accuracy was the highest.

The precision of the responses is shown in Fig 5b. The volumes of the 95% confidence ellipsoids were 284±58 cm^3^ for VS, 135±28 cm^3^ for VS+H, and 159±44 cm^3^ for VO+H. The repeated measures ANOVA again showed a significant main effect of condition (*F*_2,20_ = 7.5, *p* = 0.0038). Bonferroni-corrected post-hoc comparisons showed that only conditions VS and VS+H differed significantly (*p* = 0.030), while VO+H did not differ significantly from VS (*p* = 0.086) or VS+H (*p* = 0.93). Adding haptic guidance to the shifted visual image increased precision, which can also be seen in the typical example in Fig 4.

The level of conflict between the haptic guidance and the participant is shown in Fig 5c. It was 1.2±0.2 N for VS+H and 2.1±0.2 N for VO+H. A paired *t*-test showed a significant difference between these two conditions (*t*_10_ = −5.2, *p* < 0.001). The level of conflict was lower in the condition in which the visual image was shifted than in the condition with veridical visual information.

**Fig 7:**
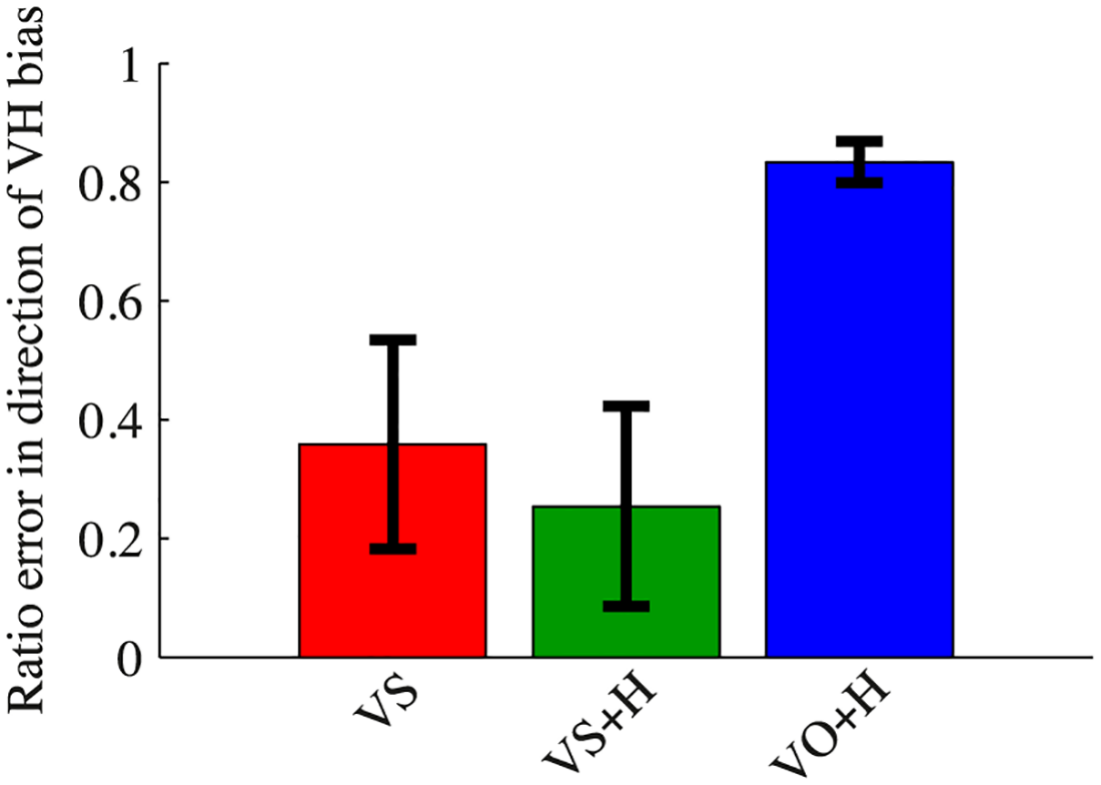
Direction of the errors in conditions VS, VS+H and VO+H. The direction is expressed as a ratio of the projection of the error vector on the vector of the visuo-haptic bias, as measured in condition VA, and the length of the error vector itself. The error vector in condition VO+H is oriented in almost the same direction as the visuo-haptic bias.

The assessment of the direction of the errors is shown in Fig 7. The analysis yielded a ratio of 0.36±0.18 for VS, 0.25±0.17 for VS+H and 0.83±0.03 for VO+H. Thus, the errors in condition VO+H were most closely aligned with the visuo-haptic biases measured in condition VA.

## Discussion

In this study, we show that adjusting for user-specific biases in haptic guidance in 3D can be useful. We used an error-based paradigm in the first condition (VA) to establish the visual positions that corresponded to the haptic representations of the target positions. In condition VS, presenting shifted visual information indeed resulted in a smaller distance between the movement end points and the target positions, and thus a better accuracy. In condition VS+H, the addition of haptic guidance towards the target locations did not further improve the accuracy (compared to condition VS), but did result in smaller confidence ellipsoids than without haptic guidance, and thus a better precision. Finally, in condition VO+H, the precision was comparable to condition VS+H, but in condition VO+H, the accuracy was poorer: the end points were further away from the targets. Together, this shows that adding haptic guidance improves precision, but correcting for visuo-haptic biases has the added benefit of also increasing accuracy. Consequently, the user-specific guidance resulted in much smaller levels of conflict between participant and guidance than did veridical information. These results are in line with the results of the previous study on user-specific adjustments of haptic guidance in 2D [17]. It is difficult to directly compare the two studies, since the biases measured in Kuling et al. were much smaller, because they were measured in a 2D-setup. When comparing the relative increase in accuracy, the previous study showed about a 20% increase in accuracy when individualizing guidance, while the current study shows about a 50% increase. However, this does not necessarily mean that one type of correcting is more useful than the other, since the setups and the absolute sizes of the biases were very different which could influence the usefulness of the individualization.

Even though the accuracy of the participants increased in VS and VS+H, the accuracy values do not have a mean of 0 for these conditions, while a perfect correction had yielded no bias in case of a sufficiently large sample size. Partly, these residual biases are caused by an imperfect adaptation in condition VA. The analysis following the experiments showed some outliers for condition VA. For the online calculation of the position of the shifted images to be presented in conditions VS and VS+H, which was based on the last 10 trials of condition VA, all the data were used. However, removing these outliers would have only slightly improved the accuracy values. Moreover, the previous study in 2D [17] also reported non-zero biases for the user-specific guidance conditions, while there was no adaptive phase present. The direction analysis can also shed some light on this problem. The results from condition VA showed that the participants’ visuo-haptic biases tended to be mainly oriented in the depth direction. Participants usually overshot the target on the first trial, after which the visual image needed to be shifted closer to them in order for them to bring their hand to the target location. This overshooting behaviour is in accordance with the literature (e.g. [14, 19, 22]). One reason for finding decreased precision in the depth direction is purely geometrical. When assuming that the targets are judged from retinal separations or changes in eye orientation, errors in angular judgements have a much larger effect in the depth direction that in the vertical plane [23]. It has been shown that for targets with limited depth information, presented in the dark, participants usually show an underestimation for the distance of far targets and an overestimation for near targets [19]. Relying on vergence cues results in increasing undershooting behaviour with increasing target distance [24]. None of our targets were presented very close to the body, and the undershooting behaviour was most prominent for the furthest targets. The direction analysis also showed that participants made errors that were close to the direction of the individual visuo-haptic biases in the condition with veridical information (VO+H). This is not surprising, since we corrected for the biases in these conditions. In the two conditions using shifted visual information, however, the directions of the errors were not aligned with the visuo-haptic biases. This again points to the residual errors in condition VS and VS+H not being directly related to the visuo-haptic biases, showing that they are not caused by an imperfect adaptation in condition VA. Thus, the origin of these residual biases remains unclear.

In a practical teleoperation situation, a shift of the visual image should be easy to implement if the visual information is provided through a screen anyway. Since the visuo-haptic biases are stable over the time span of at least one month [10], it would be sufficient to measure the biases once a month (and possibly even less often) in a simple pointing task. The biases could then be used as part of an operator-specific setup of the devices, which would determine the operator-specific shift of the visual information. An example of a system in which such an adjustment could be useful is a virtual reality based system in which hand tracking is not present, so the user’s hands are not displayed. A potential problem of adjusting for visuo-haptic biases is that, to capture the biases throughout the workspace, either a lot of points throughout the workspace or a better understanding of the mechanism of the biases would be needed. If future research would provide us with a model of these biases, it would be far easier to make the proper adjustment throughout the workspace or even predict biases by modelling them for the specific user.

This study shows that individualizing guidance results in a higher accuracy and a lower level of conflict between operator and guidance than traditional, veridical guidance does. So, perceptually consistent haptic guidance seems to have benefits over physically correct guidance. In addition to these immediate benefits, we think that the added benefit of reducing the amount of conflict is that this probably increases the operator’s trust in the system, since the system is more intuitive to use. It has been shown that intuitiveness is a very important factor in the usefulness of haptic guidance [25]. When the same amount of information is present, a more intuitive type of haptic guidance results in a better operator performance. This means that user-specific adjustments to haptic guidance show potential to increase operator performance, decrease the amount of conflict between operator and machine and make the contact between operator and machine more intuitive.

## Conclusions

In this study, we have investigated the usefulness of correcting for user-specific biases in human perception in the design of haptic guidance. As an example of user-specific biases, we used the paradigm of visuo-haptic biases. Our data show that user-specific guidance increased accuracy and decreased the amount of conflict between operator and machine. The precision was improved in all conditions in which haptic guidance was presented. These results show the potential of correcting for user-specific perceptual biases when designing haptic guidance.

## Supporting Information

S1 File Position and force data recorded with the Haptic Master. Filenames indicate the participant (‘pp*’) and the condition (‘cond*’). Condition 1 is condition VA, condition 2 is condition VS, condition 3 is condition VS+H, and condition 4 is condition VO+H.
(ZIP)

S2 File Position data recorded with the Optotrak, based on IRED tracking. Filenames indicate the participant (‘pp*’) and the condition (‘cond*’). Condition 1 is condition VA, condition 2 is condition VS, condition 3 is condition VS+H, and condition 4 is condition VO+H.
(ZIP)
